# Peripheral nerve injury-induced alterations in VTA neuron firing properties

**DOI:** 10.1186/s13041-019-0511-y

**Published:** 2019-11-04

**Authors:** Shuo Huang, Stephanie L. Borgland, Gerald W. Zamponi

**Affiliations:** 1 Department of Physiology and Pharmacology, Hotchkiss Brain Institute, Calgary, AB Canada; 2 Children’s Hospital Research Institute, Cumming School of Medicine, University of Calgary, Calgary, AB Canada

**Keywords:** Dopamine, Ventral tegmental area, Pain, Prefrontal cortex, Brain circuits

## Abstract

The ventral tegmental area (VTA) is one of the main brain regions harboring dopaminergic (DA) neurons, and plays important roles in reinforcement and motivation. Recent studies have indicated that DA neurons not only respond to rewarding stimuli, but also to noxious stimuli. Furthermore, VTA DA neurons undergo plasticity during chronic pain. Lateral and medial VTA neurons project to different brain areas, and have been characterized via their distinct electrophysiological properties. In this study, we characterized electrophysiological properties of lateral and medial VTA DA neurons using DAT-cre reporter mice, and examined their plasticity during neuropathic pain states. We observed various DA subpopulations in both the lateral and medial VTA, as defined by action potential firing patterns, independently of synaptic inputs. Our results demonstrated that lateral and medial VTA DA neurons undergo differential plasticity after peripheral nerve injury that leads to neuropathic pain. However, these changes only reside in specific DA subpopulations. This study suggests that lateral and medial VTA DA neurons are differentially affected during neuropathic pain conditions, and emphasizes the importance of subpopulation specificity when targeting VTA DA neurons for treatment of neuropathic pain.

## Introduction

Dopaminergic (DA) neurons within the ventral tegmental area (VTA) play an important role in the regulation of appetitive stimuli, anxiety, aversion and pain [1–5]. The precise function of VTA DA neurons in pain processing is incompletely understood. It has been suggested that pain relief is signaled as reward via VTA DA neurons [6]. However, VTA DA neurons are also directly activated by noxious stimuli [7]. Recent studies revealed plasticity of VTA dopamine neurons during neuropathic pain, expressed as decreased excitability [8, 9]. Notably, noxious stimuli, such as footshocks, induce phasic firing in only specific subpopulations of VTA DA neurons [6, 7]. However most, if not all existing studies reported plasticity in VTA DA neurons in chronic pain conditions as a whole population. VTA DA neurons are heterogeneous. They receive inputs from, and project to a number of brain regions, including the nucleus accumbens (NAc), the medial prefrontal cortex (mPFC), and the amygdala [10–12], which have been implicated in the processing of both reward and pain [13–15]. While some subpopulations can be separated depending on their locations (i.e., NAc lateral and medial shell projecting neurons are mainly located in the lateral and medial VTA, respectively), others are intermingled within the same VTA subregion (mPFC, amygdala and NAc core projecting neurons are located mainly in the medial VTA) [16–19].

Here, we examined biophysical properties and neuronal excitability of VTA DA neurons in SHAM operated mice, and in mice with a spared nerve injury (SNI) of the sciatic nerve which gives rise to chronic neuropathic pain [14, 20]. In both the lateral and medial VTA, we used action potential firing pattern as an electrophysiological fingerprint to define different DA subpopulations. We find that only specific subtypes of medial and lateral dopaminergic VTA undergo injury-induced changes in their electrophysiological properties, thus suggesting that chronic pain states are associated with altered neuronal plasticity in specific DA neuron populations in the VTA.

## Methods

### Animals

All experiments involving animals were performed under the Canadian Council on Animal Care Committee Guidelines, and were approved by the University of Calgary Animal Care Committee. Animals were maintained under a 12 h light/dark cycle with free access to food and water. Male adult DAT-Ires-Cre x Ai9 mice were used for experiments. These mice were generated by crossing a B6.SJL-Slc6a3tm1.1(cre)Bkmn/J mouse line (DAT-Ires-Cre) with a B6.Cg-Gt(ROSA)26Sor < tm9(CAG-tdTomato)Hze>/J conditional allele mouse line (Ai9) (Jackson Laboratories) to express tdTomato under the DAT promoter. DAT is a specific marker for DA neurons [21, 22], and has been extensively characterized and widely used in previous studies [23, 24]. All animals were subjected to SNI/SHAM surgeries at 6 weeks and used for electrophysiology recordings between 8 to 11 weeks old.

### Electrophysiology

Mice were decapitated under anesthesia. The brain was removed from the skull and immediately placed into ice-cold, oxygenated NMDG solution (159.2 mM NMDG, 74.6 mM KCl, 1.2 mM NaH_2_PO_4_, 30 mM NaHCO_3_, 20 mM HEPES, 25 mM D-glucose, 5 mM Na-ascorbate, 3 mM Na-pyruvate, 10 mM MgSO_4_.7H_2_O, 2 mM Thiourea, and 0.5 mM CaCl_2_.2H_2_O, prepared in MilliQ H_2_O, pH = 7.4) for 2 min. Horizontal sections of 260 μm through the VTA were cut with a vibratome (Leica VT 1200S) at room temperature, incubated in NMDG solution for 12 min at 33 °C, transferred into extracellular recording solution (119 mM NaCl, 26 mM NaHCO_3_, 25 mM Glucose, 2.5 mM KCl, 1.25 mM NaH_2_PO_4_, 2.5 mM CaCl_2_, and1.3 mM MgSO_4_, prepared in MilliQ H2O, pH = 7.4), and incubated for another 45 min at 33 °C. Whole-cell patch clamp recordings were carried out on VTA dopaminergic cells using a (MultiClamp 700B amplifier (Molecular Devices) and a digitizer (Digidata 1440A, Molecular Devices) to investigate synaptic transmission and cell excitability properties. Dopaminergic neurons were identified through expression of tdTomato. Cell excitability properties were examined using current-clamp recording, with glass pipettes (4–6 MΩ) filled with physiological intracellular recording solution (130 mM K-gluconate, 10 mM HEPES, 0.2 mM EGTA, 10 mM Na_2_-phosphocreatine, 4 mM Mg-ATP, and 0.3 mM Na-GTP, 1.3 mM biocytin, PH = 7.2). Cell excitability properties include spontaneous firing, frequency-current (F-I) relationship, action potential threshold, input resistance, Ih, and membrane potential. Membrane potential was read when holding current was 0 pA after cells were stabilized. Cells were then held at − 80 mV for measurements of other excitability properties. The F-I relationship was determined by using a series of 1 s 25 pA current steps starting from 0 pA, and data were plotted as number of spikes per second at different injecting current levels. To study cell input resistance, cells were injected using a series of 1 s − 25 pA current steps starting from 0 pA. Input resistance was determined by dividing the membrane potential of each trace by its injecting current. Ih was calculated by dividing voltage sag by input resistance. The voltage sag was calculated by subtracting peak response and steady-state potential at a − 150 pA hyperpolarizing step [25, 26]. Voltage threshold of action potentials was measured using a 100 ms depolarizing current ramp of 40–60 pA. All data were digitized at 20 kHz and filtered at 10 kHz. For blocker experiments, 20 μM Bicuculline, 50 μM D-AP5, 10 μM DNQX, 500 μM Sulpiride, 200 nM CGP55845, 1 μM Strychnine were bath perfused after recording of excitability properties for 5 min. Excitability properties were then collected again with blockers. A junction potential of 15 mV (calculated using pClamp 10, Molecular Devices) was subtracted from all membrane potentials, including cell membrane potential, holding potential, and action potential threshold.

### Immunohistochemistry

Immunohistochemistry was carried out on free floating sections after patching to confirm recording locations. Immediately after patch clamp recordings, VTA sections were fixed using 4% paraformaldehyde, and kept at 4 °C up to a month before staining. Brain sections were washed using 1X PBS before being blocked with vehicle (0.3% TritonX-100, 10% normal goat serum, 0.5% bovine serum albumin) for 1.5 h at room temperature, and incubated with conjugated antibody (200–542-211, Jackson ImmunoResearch, PA, USA) overnight at 4 °C. Slides were then washed with vehicle, 1X PBS, and 0.5X PBS before being placed and air dried on a slice, and cover-slipped with mounting media (Sigma). Images were taken with a confocal microscope (Leica LAS).

### Spared nerve injury (SNI) neuropathic pain model

Adult mice were placed under anesthesia. Three terminal branches (tibial, common peroneal, and sural nerves) of the sciatic nerve in the left hind leg were identified. Tibial and common peroneal nerves were tied using a 6.0 suture, cut, and left disconnected. At the open end at which the suture was tied, 1 mm of the nerve was removed. The sural nerve was left intact. For SHAM operated mice, three terminal branches of the sciatic nerve were identified but not touched before the incision on the skin was closed. Emla cream was used as local anesthetic to reduce post-surgery pain. Animals were monitored after surgery to ensure proper wound healing.

### Statistics

Data were analyzed using OriginPro 9.1 for comparisons, and GraphPad QuickCalcs for exclusion of outliers. For comparison of spontaneous firing properties, all non-firing neurons were excluded. All comparisons between two groups including lateral versus medial VTA, and SHAM versus SNI were done with Mann-Whitney tests, and data in text were presented as mean ± SEM. Comparisons between three groups were done with one-way ANOVA, and a Bonferroni post-hoc test. Statistical significance was reported when *p* < 0.05. In all figures, * represents *p* < 0.05, ** represents *P* < 0.01, *** represents *P* < 0.001, **** represents *P* < 0.0001. Numbers of experiments are presented as (*N* = cells/*N* = animals).

## Results

### Nerve injury-induced changes in the total medial and lateral VTA neuron population

**Fig. 1:**
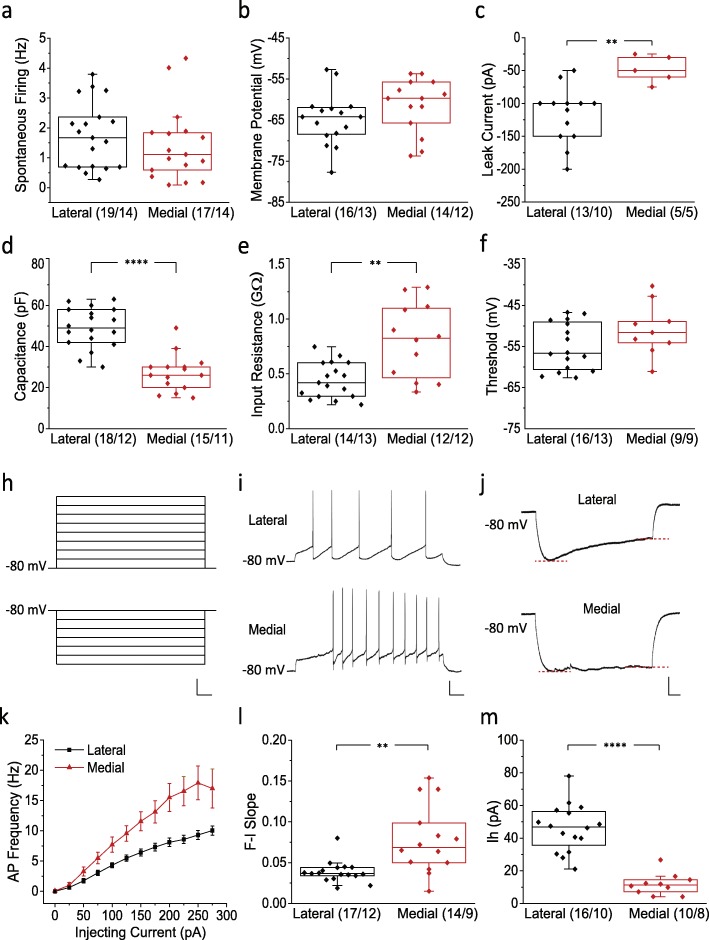
Electrophysiological characterization of lateral and medial VTA DA neurons. **a** Spontaneous firing frequencies in the lateral and medial VTA. **b-f** Biophysical properties including membrane potential (**b**), leak current (**c**), capacitance (**d**), input resistance (**e**), and action potential threshold (**f**) of medial and lateral VTA DA neurons. **h** Current clamp stimulation protocols for measuring the F-I relation (upper) and Ih (lower). *Scale bar, 50 mV, 100 ms*. **i** Representative current clamp traces showing AP firing patterns at 100 pA from lateral (upper) and medial (lower) VTA DA neurons. *Scale bar, 20 mV, 100 ms*. **j** Representative current clamp traces showing activation of Ih in lateral (upper) and medial (lower) VTA DA neurons. The red dashed lines indicate where peak response and steady-state were measured for calculation of the voltage sag. *Scale bar, 10 mV, 100 ms*. **k** Average F-I relation curves for medial and lateral VTA DA neurons. **l** F-I slope for medial and lateral VTA DA neurons. **m** Ih amplitude recorded from medial and lateral VTA DA neurons. Statistics, Mann-Whitney test. **, *p* < 0.01; ****, *p* < 0.0001. Numbers in parentheses reflect numbers of cells and animals, and are presented as (*N* = cells/*N* = animals)

We performed recordings in slices from the lateral and medial VTA to ascertain putative plasticity in DA neurons as a result of peripheral nerve injury (leading to neuropathic pain). Prior to comparing neuronal excitability in SNI to that in SHAM operated mice, electrophysiological properties were characterized in lateral and medial VTA DA neurons that were identified by tdTomato fluorescence driven by the DAT promoter (Additional file 1: Figure S1). Consistent with previous literature [27], while some features were similar between lateral and medial VTA (Fig. 1a-c, f), we found that lateral VTA neurons are larger in size (49.06 ± 2.32 pF, *n* = 18/12) compared to medial VTA neurons (27.07 ± 2.32 pF, *n* = 15/11)(*p* = 1.40E-5, Mann Whitney test), as reflected by cell capacitance [28] (Fig. 1d). This was associated with a bigger leak current (lateral − 117.31 ± 11.83 pA, *n* = 13/10; medial 48.00 ± 9.30 pA, *n* = 5/5;*p* = 0.0052, Mann Whitney test) as well as a smaller input resistance (lateral 0.44 ± 0.04 GΩ, *n* = 14/13; medial 0.80 ± 0.10 GΩ, *n* = 12/12; *p* = 0.0051, Mann Whitney test) in lateral VTA neurons (Fig. 1c and e), which result in a lower excitability as reflected in the F-I slope (lateral 0.039 ± 0.003, *n* = 17/12; medial 0.078 ± 0.011, *n* = 14/9; *p* = 0.034, Mann Whitney test) (Fig. 1i, k, l). Another well-documented electrophysiological fingerprint of lateral VTA versus medial is activation of the hyperpolarization-activated cation current (Ih) [27], which was also observed in our recordings (lateral 46.07 ± 3.62 pA, *n* = 16/10; medial 11.80 ± 2.10 pA, *n* = 10/8; *p* = 3.51E-5,Mann Whitney test) (Fig. 1j and m).

**Fig. 2:**
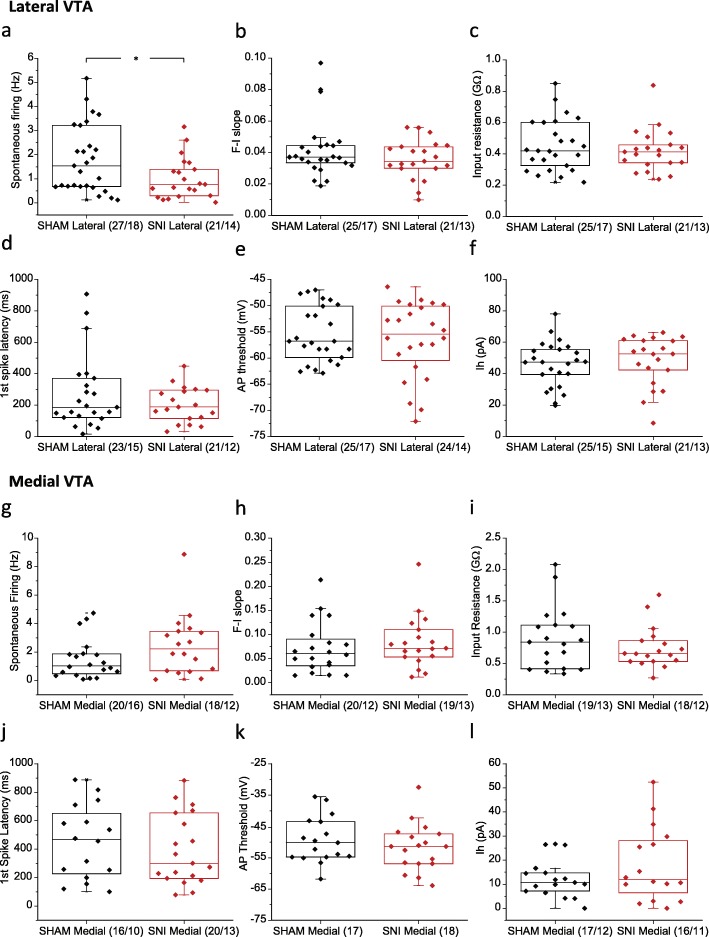
Electrophysiological properties of lateral and medial VTA DA neurons isolated from in SHAM versus SNI groups. **a-f** Compariaon of spontaneous firing frequency (**a**), F-I slope (**b**), input resistance (**c**), first spike latency at 100 pA (**d**), AP threshold (**e**), and Ih amplitude (f) between SHAM and SNI groups in lateral VTA DA neurons. **g-l** Comparison of spontaneous firing frequency (**g**), F-I slope (**h**), input resistance (**i**), first spike latency at 100 pA (**j**), AP threshold (**k**), and Ih amplitufe (**l**) between SHAM and SNI groups in medial VTA DA neurons. Statistics, Mann-Whitney test. *, *p* < 0.05. Numbers in parentheses reflect numbers of cells and animals, and are presented as (*N* = cells/*N* = animals)

After confirming the quality and location of our recordings, biophysical and excitability properties were recorded in DA neurons from mice that had undergone SNI surgeries, and compared with those in SHAM groups. The SNI model was validated by pain behavioural tests (Additional file 2: Figure S2), using a different set of mice than the ones used for electrophysiology test, to avoid putative CNS changes that might be induced by acute noxious stimuli. In the lateral VTA, SNI surgery induced a decrease in spontaneous firing frequency (SHAM 1.81 ± 0.27 Hz, *n* = 27/18; SNI 1.01 ± 0.19 Hz, *n* = 21/14; *p* = 0.037, Mann Whitney test) (Fig. 2a), but it did not affect other properties including F-I slope, input resistance, first spike latency, action potential threshold and Ih (Fig. 2b-f). In the medial VTA, no difference was observed between SNI and SHAM operated mice in the properties that were examined (Fig. 2g-l).

### Injury induced changes in the firing behavior of lateral VTA neuron subtypes

**Fig. 3:**
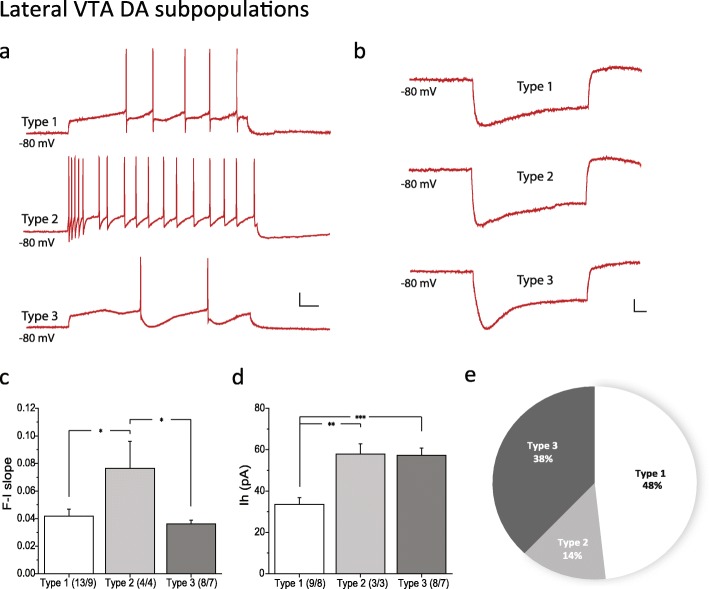
Electrophysiological characterization of three subpopulations of lateral VTA DA neurons. **a** Examples of AP firing patterns evoked by a 100 pA current step in Type 1–3 DA neurons. **b** Ih activation evoked by a − 150 pA current step in Type 1–3 DA neurons. **c** Quantification of F-I slope in Type 1–3 DA neurons. **d** Quantification of Ih amplitude in Type 1–3 DA neurons. **e)** Proportion of Type 1–3 neuronal subtypes in the lateral VTA DA neurons. *Scale bar, 20 mV, 100 ms*. Statistics, One-way ANOVA and Bonferroni post-hoc test. *, *p* < 0.05; **, *p* < 0.01; ***, *p* < 0.001. Numbers in parentheses reflect numbers of cells and animals, and are presented as (*N* = cells/*N* = animals). Characterization was done with SHAM operated mice

Because we observed SNI-induced plasticity only in lateral VTA DA neurons, we hypothesized that specific sub-populations within the lateral VTA DA may be more susceptible than others in response to SNI, given the heterogeneity within the VTA [16–18]. Based on action potential firing patterns observed during evoked action potential recordings (see paradigm in Fig. 1h, upper panel), lateral VTA DA neurons were grouped into Types 1–3 (Fig. 3a). Type 1 neurons have a delayed onset of first spike and a regular (nonaccommodating) firing pattern. Type 2 neurons fire immediately at the start of current stimulation. The firing frequency adapts and the excitability reflected by the F-I slope is significantly higher compared to the other 2 types (Type 1, 0.041 ± 0.005, *n* = 13/9; Type 2, 0.076 ± 0.019, *n* = 4/4; Type 3, 0.036 ± 0.003, *n* = 8/7; *p* = 0.010, one-way ANOVA; *p* = 0.021 Type 1 vs. 2, *p* = 0.012 Type 2 vs. 3, *p* = 1 Type 1 vs. 3, Bonferroni test) (Fig. 3c). Type 3 neurons exhibit a similar firing pattern as Type 1 neurons, but exhibit a much larger and slower after-hyperpolarization potential (AHP). In addition, Type 2 and 3 neurons have a significantly bigger Ih compared to Type 1 neurons (Type 1, 33.5 ± 3.3 pA, *n* = 9/8; Type 2, 57.9 ± 4.9 pA, *n* = 3/3; Type 3, 57.3 ± 3.5 pA, *n* = 8/7; *p* = 1.61E-4, one-way ANOVA; *p* = 0.0044 Type 1 vs. 2, *p* = 1 Type 2 vs. 3, *p* = 2.82E-4 Type 1 vs. 3, Bonferroni test) (Fig. 3d), but they differ in the kinetics of Ih activation (Fig. 3b). These characteristic firing patterns were maintained in the presence of synaptic blockers (bicuculline 20 μM, D-AP5 50 μM, DNQX 10 μM, sulpiride 500 μM, CGP55845 200 nM, strychnine 1 μM) that inhibit GABA_A_, NMDA, AMPA, D2, GABA_B_, and glycine receptors, respectively, indicating that these firing patterns are intrinsic (Additional file 3: Figure S3a-c). For all three DA subpopulations in the lateral VTA, application of antagonists increased action potential frequency at an identical stimulation current step, but did not change action potential characteristics. This is consistent with previous findings that VTA DA neurons are constrained by inhibitory tone [29–31].

**Fig. 4:**
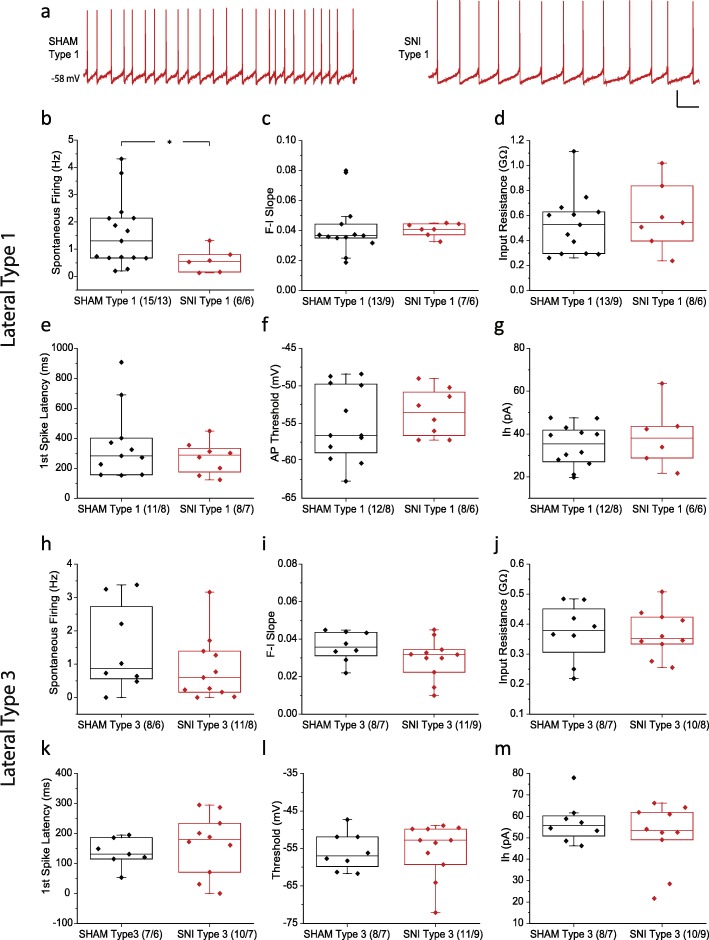
Electrophysiological properties of Type 1 and 3 lateral VTA DA neuron subpopulations in SHAM and SNI groups. **a-f** (**a**) Representative traces of spontaneous firing of Type 1 neurons in SHAM (left) and SNI (right) operated mice. *Scale bar, 20 mV, 1 s*. Comparison of spontaneous firing frequency (**b**), F-I slope (**c**), input resistance (**d**), first spike latency at 100 pA (**e**), AP threshold (**f**), and Ih amplitude (**g**) between SHAM and SNI groups in Type 1 lateral VTA DA neurons. **h-m** Comparison of spontaneous firing frequency (**h**), F-I slope (**i**), input resistance (**j**), first spike latency at 100 pA (**k**), AP threshold (**l**), and Ih amplitude (**m**) between SHAM and SNI groups in Type 3 lateral VTA DA neurons. Statistics, Mann-Whitney test. *, *p* < 0.05. Numbers in parentheses reflect numbers of cells and animals, and are presented as (*N* = cells/*N* = animals)

The majority of lateral DA neurons belong to Type 1 (28/58) and 3 (22/58) (Fig. 3e). We therefore compared electrophysiological properties between SHAM and SNI in these two groups (Fig. 4). Our results demonstrated that the nerve injury-induced decreased spontaneous firing frequency that was observed in the total lateral DA population (see Fig. 2) is due to changes in the properties of Type 1 (SHAM 1.56 ± 0.32 Hz, *n* = 15/13; SNI 0.59 ± 0.18 Hz, *n* = 6/6; *p* = 0.047, Mann Whitney test)(Fig. 4a, b) but not Type 3 neurons (Fig. 4h), while all other properties in both groups remain unchanged after SNI surgery.

Lateral VTA DA neurons were also compared at the ventral-dorsal axis. Lateral central VTA DA neurons have a higher excitability compared to those in the lateral ventral region, as reflected in the F-I slope (central 0.054 ± 0.008, *n* = 20/17; ventral 0.034 ± 0.003, *n* = 17/13; dorsal 0.033 ± 0.004, *n* = 9/8; *p* = 0.021, one-way ANOVA; *p* = 0.0039, Bonferroni post-hoc test) (Additional file 4: Figure S4). However, no difference between SHAM and SNI operated groups was observed in the ventral, central, nor dorsal lateral VTA (data not shown).

### Injury induced changes in the firing behavior of medial VTA neuron subtypes

**Fig. 5:**
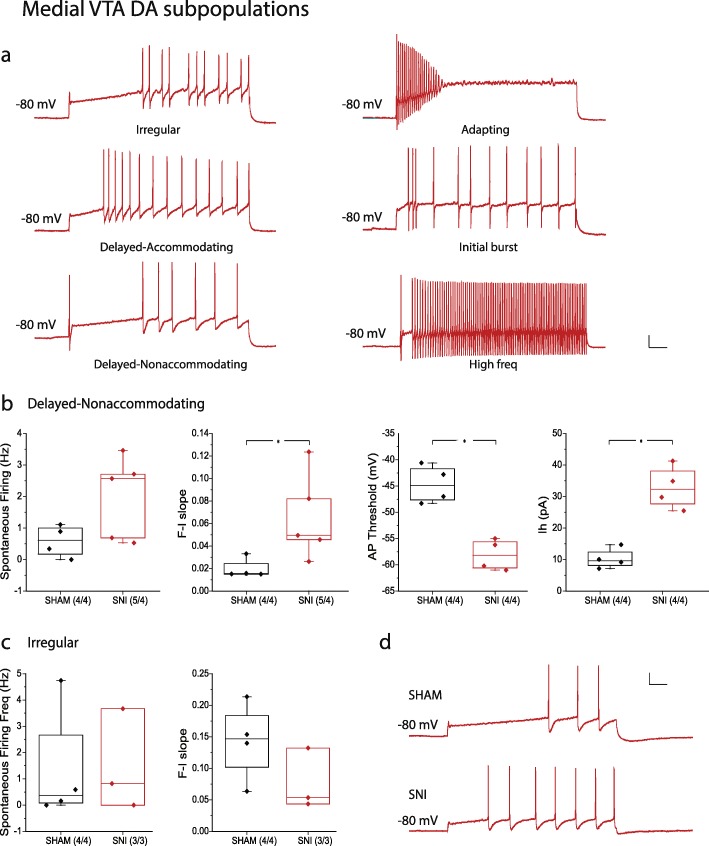
Electrophysiological properties of DA neuronal subpopulations in the medial VTA in SHAM and SNI groups. **a** Examples of AP firing patterns evoked by a 200 pA current step of different DA neuronal subpopulations in the medial VTA. **b** Electrophysiological properties of *Delayed-nonaccommodating firing type* DA neurons in SHAM versus SNI groups. **c** Electrophysiological properties of *Irregular firing type* DA neurons in SHAM versus SNI groups. **d** Representative current clamp traces showing the number of APs during a 150 pA current step in SHAM (upper) and SNI (lower) groups. *Scale bar, 20 mV, 100 ms*. Statistics, Mann-Whitney test. *, *p* < 0.05. Numbers in parentheses reflect numbers of cells and animals, and are presented as (*N* = cells/*N* = animals)

Different DA subpopulations were also observed in the medial VTA (Fig. 5). DA neurons were grouped into subpopulations based on their firing patterns, using the following criteria which are widely used for classification of cortical neurons and neurons in other brain areas [32–35]: 1) accommodating, nonaccommodating, bursting, and irregular spiking; 2) delayed and classic; 3) initial, transient, and repetitive. The observed firing patterns include, but are not limited to *irregular* (8/42), *delayed-accommodating* (3/42), *delayed-nonaccommodating* (9/42), *adapting* (3/42), *initial burst* (2/42), and *high frequency* (firing rate above 35 Hz at + 200 pA current step) subtypes types (2/42) (Fig. 5a). Among these, the responses of *irregular*, *delayed-nonaccommodating*, and *high frequency* neurons to synaptic blockers were tested, revealing a drug-induced increase in excitability, without a change in their overall firing patterns (Additional file 3: Figure S3g-i). This suggests that specific firing patterns in medial VTA DA neurons are an intrinsic feature that is independent from synaptic input. The majority of the observed medial VTA DA neuron subtypes were of the *irregular* and *delayed-nonaccommodating* phenotypes, and we compared electrophysiological properties between SHAM and SNI of these two subpopulations (whereas the others were observed too infrequently to permit a comparison between SHAM and SNI states). In contrast with lateral VTA Type 1 neurons, the *delayed-nonaccommodating* firing medial VTA neurons from SNI treated mice showed a higher degree of excitability (as reflected by the F-I slope) compared to the SHAM group (SHAM 0.020 ± 0.004, *n* = 4/4; SNI 0.065 ± 0.017, *n* = 5/4; *p* = 0.037, Mann Whitney test) (Fig. 5b and d). Consistently, we observed a more hyperpolarized action potential threshold (SHAM − 44.68 ± 1.80 mV, *n* = 4/4; SNI − 58.10 ± 1.47 mV, *n* = 4/4; *p* = 0.030, Mann Whitney test) and larger Ih amplitude (SHAM 10.28 ± 1.60 pA, *n* = 4/4; SNI 32.85 ± 3.40 pA, *n* = 4/4; *p* = 0.030, Mann Whitney test) in the SNI group. There was no change in spontaneous firing frequency (Fig. 5b) and in other biophysical properties (data not shown) in response to SNI surgery. In contrast, the *irregular* firing medial VTA neurons did not exhibit a difference in cell excitability, spontaneous firing frequency (Fig. 5c), as well as other biophysical properties (data not shown). Medial VTA DA neurons were also compared at the ventral-dorsal axis. However no electrophysiological fingerprint could be identified based only on ventral-dorsal locations (Additional file 4: Figure S4), and no difference in electrophysiological properties between SHAM and SNI operated groups was observed in ventral, central, nor dorsal medial VTA (data not shown). Altogether, these data indicate that nerve injury alters the firing properties of specific subpopulations of VTA DA neurons.

## Discussion

The role of VTA DA neurons in motivated behaviour and conditioned reinforcement has been well documented. However, their functions in pain or aversion signaling have remained controversial [6, 7] and incompletely understood. Existing studies using electrophysiology [8, 9] or indirect measurements by microdialysis [36] indicate that chronic pain states are associated with a hypodopaminergic tone in the VTA, while noxious stimuli are signaled as motivational salience and trigger dopamine release [37]. Chronic pain is different from acute noxious stimuli, and may involve different mechanisms and neuronal subpopulations in the VTA. In the present study, we provide new evidence that the overall activity of DA neurons in the lateral, but not the medial VTA is decreased after peripheral nerve injury. Furthermore, we dissected DA neurons into different subpopulations based on their firing patterns. Our results revealed that plasticity induced during neuropathic pain states only resides in a single specific DA subpopulation in both the lateral and medial VTA. The changes in *delayed-nonaccommodating* firing medial VTA neurons might be masked by other unchanged subtypes, due to the complexity of medial VTA DA populations as shown here as well as in previous studies [17].

VTA DA neurons send projections to the NAc and the mPFC via mesolimbic and mesocortical pathways. These two limbic areas have been intensively studied in the context of pain perception and modulation, and DA has been suggested to play a role in these processes. Thus, changes in VTA DA neurons may be further reflected in their downstream targets [8, 38–40]. Zhang *et. al.* reported that peripheral nerve injury (CCI) increased spontaneous firing frequency in VTA-NAc neurons. The change in neuronal activity in the VTA can further increase BDNF expression in the NAc, which has a causal relation to neuropathic pain [38]. The authors did not clearly state which subregion of the NAc they focused on. Given that the authors reported an increase in firing rate, our results may suggest that their observed changes might have occurred in VTA neurons that might project to the medial shell of the NAc. As another example, changed VTA DA neuron activity can affect indirect pathway projections to spiny neurons in the NAc, which is known to have a causal relation to neuropathic pain [8]. However, compared to an overall decrease in medial VTA neurons reported in this study, our results showed an increase in the *delayed-nonaccommodating* firing subtype and no overall change in the medial VTA. The different results might be due to different experimental conditions. For example, we used DAT-reporter mice to specifically identify DA neurons, and we did not use TTX to block action potential firing during recordings. This difference might indicate that nerve injury-induced changes may affect VTA DA populations by both synaptic input and intrinsic properties. In addition, changes in VTA DA neuronal activity may modulate PFC activity via the mesocortical pathway, thus affecting pain perception through the PFC-PAG axis [39, 41, 42].

Although our study reveals nerve injury-induced plasticity in the VTA, it is still unclear whether this change is causal to chronic neuropathic pain. Previous studies reported that lesion of DA neurons in VTA and terminals in the striatum using 6-OHDA increased hyperalgesia during by neuropathic pain conditions [43]. In contrast, electrical stimulation in the VTA has analgesic effects [44, 45]. Moreover, stimulating NAc projecting VTA DA neurons reverses neuropathic pain [9]. It is important to note that in the present study, the two groups of neurons that showed nerve injury-induced plasticity could only be identified based on their firing pattern. Further experiments using retrograde tracing techniques to investigate the input/output map will be needed to further dissect the circuitry.

In summary, our data reveal that peripheral nerve injury alters the activity of specific subpopulations of VTA neurons. Whereas, the molecular basis for the observed changes will require further study, our data suggest targeting the VTA as a possible locus for intervention into neuropathic pain.

## Supplementary information

**Additional file 1: Figure S1.** *Biocytin labeling in the internal recording solution allows post-hoc recovery of recording locations.* **a**) VTA horizontal section showing recorded neurons from the medial (left arrow) and lateral (right arrow) VTA. Red, DAT-positive neurons expressing td-Tomato. Green, biocytin label*. Scale bar, 100* μm*.* **b**) and **c**) are images of the same neurons with and without biocytin signal, showing morphological details of cells and the overlap between DAT and biocytin. *Scale bar, 50 μm.*

**Additional file 2: Figure S2**. *Mechanical withdrawal threshold in SNI and SHAM mice.* **a**) Mechanical withdrawal threshold of ipsilateral (ipsi) and contralateral (contra) paws in 10 SNI operated mice. **b**) Mechanical withdrawal threshold of ipsilateral (ipsi) and contralateral (contra) paws in 6 SHAM operated mice. Numbers in parentheses reflect numbers of animals.

**Additional file 3: Figure S3.** *AP firing patterns of different DA neuronal subpopulations in the lateral and medial VTA without and with synaptic blockers.* **a-c**) Examples of AP firing patterns of lateral VTA Type 1–3 neurons evoked by a 100 pA current step, without blockers. **d-f)** AP firing patterns of lateral VTA Type 1–3 neurons evoked by a 100 pA current step in the presence of synaptic blockers (Bicuculline 20 μM, D-AP5 50 μM, DNQX 10 μM, Sulpiride 500 μM, CGP55845 200 nM, Strychnine 1 μM). **g-i**) AP firing patterns of medial VTA *Irregular*, *Delayed-Nonaccommodating*, and *High frequency* DA neuron subpopulations at a 150 pA current step in the absence of synaptic blockers. **j-l**) AP firing patterns of medial VTA *Irregular*, *Delayed-Nonaccommodating,* and *High frequency* DA neuron subpopulations at 150 pA current step, with the above synaptic blockers. *Scale bar, 20 mV, 100 ms*.

**Additional file 4: Figure S4.** *Electrophysiological properties of ventral-dorsal VTA DA neurons.* **a-d**) Spontaneous firing frequency (**a**), F-I slope (**b**), input resistance (**c**), and Ih (**d**) in central, ventral, and dorsal subregions of the lateral VTA. **e-h**) Spontaneous firing frequency (**e**), F-I slope (**f**), input resistance (**g**), and Ih (**h**) in central, ventral, and dorsal subregions of the medial VTA. Numbers in parentheses reflect numbers of cells. DATA were collected from 33 SHAM and 25 SNI mice.

## Data Availability

The data used in our study are available from the authors on reasonable request.

## Abbreviations

AP: Action potential
BDNF: Brain-derived neurotrophic factor
CCI: Chronic constriction injury
DA: Dopamine
DAT: Dopamine transporter
mPFC: medial prefrontal cortex
NAc: Nucleus accumbens
SEM: Standard error of the mean
SNI: Spared nerve injury
VTA: Ventral tegmental area
