# Supplementary figures and images for: Peripheral nerve injury-induced alterations in VTA neuron firing properties
Source: Mol Brain. 2019 Nov 4;12:89. doi: 10.1186/s13041-019-0511-y (PMC6827252; doi:10.1186/s13041-019-0511-y)

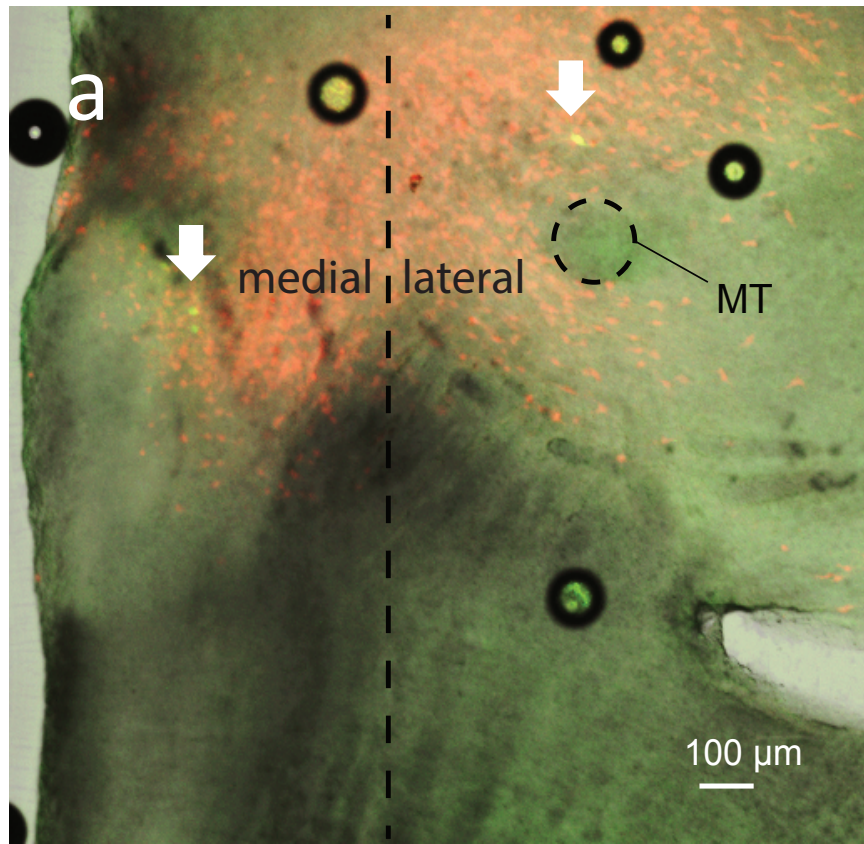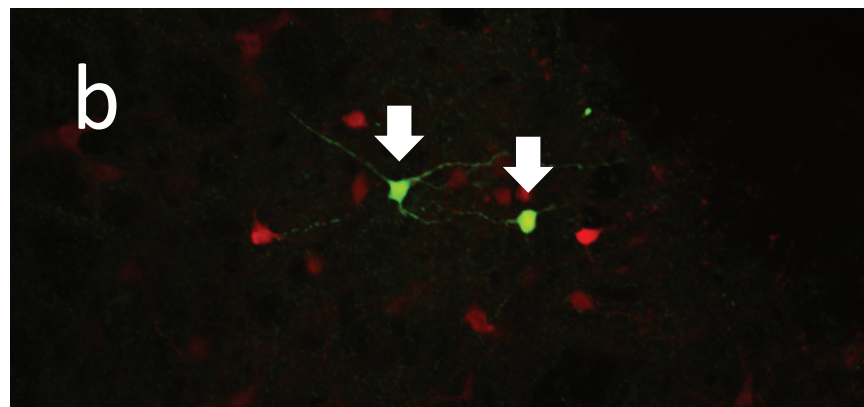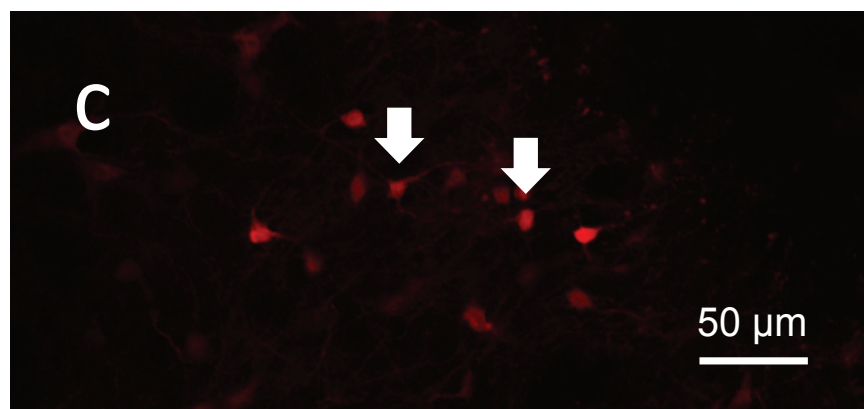

Supplement: Supplementary file 1 — Additional file 1: Figure S1. Biocytin labeling in the internal recording solution allows post-hoc recovery of recording locations. a) VTA horizontal section showing recorded neurons from the medial (left arrow) and lateral (right arrow) VTA. Red, DAT-positive neurons expressing td-Tomato. Green, biocytin label. Scale bar, 100 μm. b) and c) are images of the same neurons with and without biocytin signal, showing morphological details of cells and the overlap between DAT and biocytin. Scale bar, 50 μm. [file 13041_2019_511_MOESM1_ESM.pdf]

a

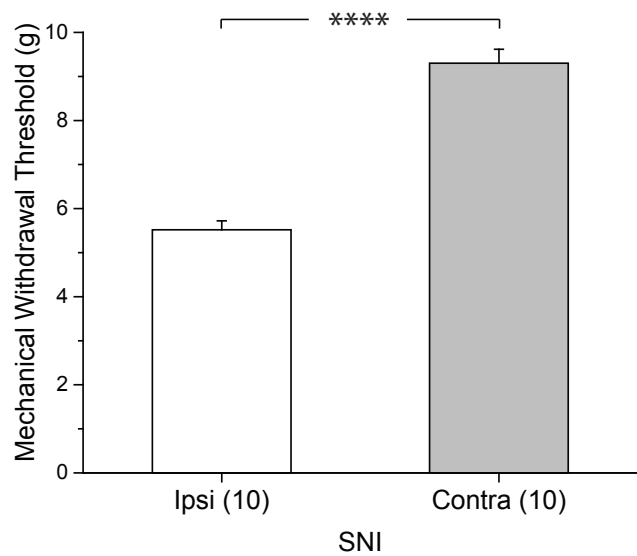

b

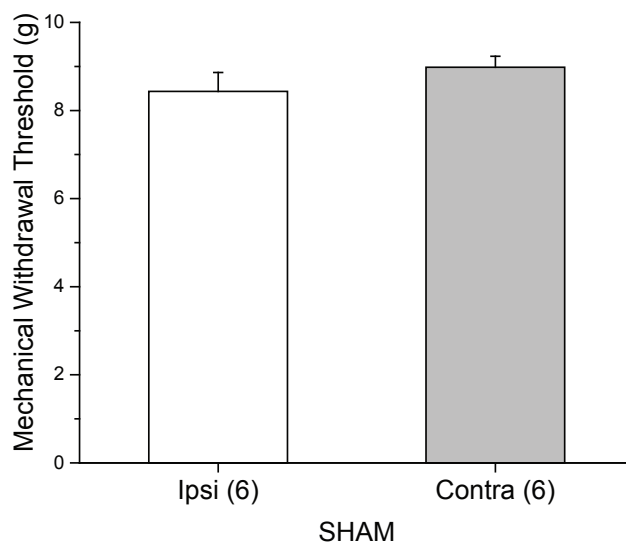

Supplement: Supplementary file 2 — Additional file 2: Figure S2. Mechanical withdrawal threshold in SNI and SHAM mice. a) Mechanical withdrawal threshold of ipsilateral (ipsi) and contralateral (contra) paws in 10 SNI operated mice. b) Mechanical withdrawal threshold of ipsilateral (ipsi) and contralateral (contra) paws in 6 SHAM operated mice. Numbers in parentheses reflect numbers of animals. [file 13041_2019_511_MOESM2_ESM.pdf]

Lateral VTA

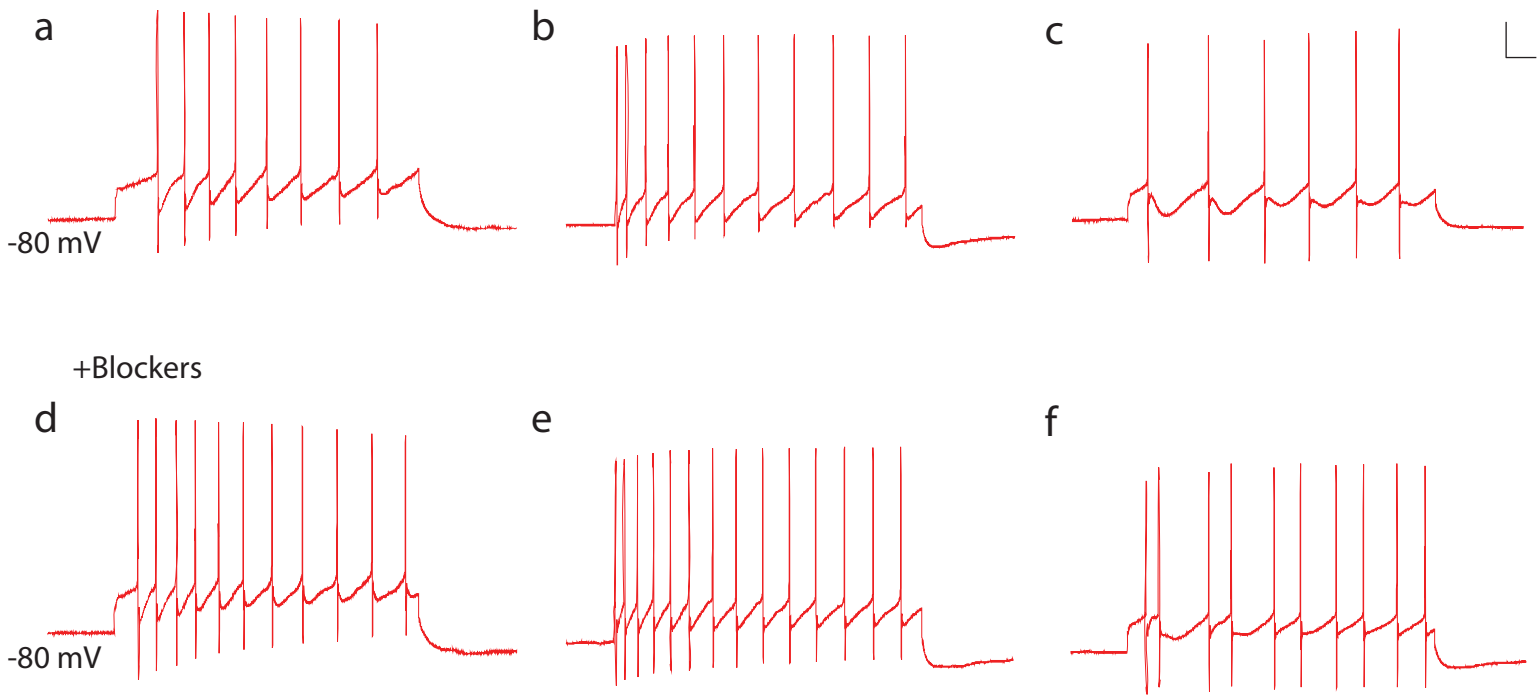

Medial VTA

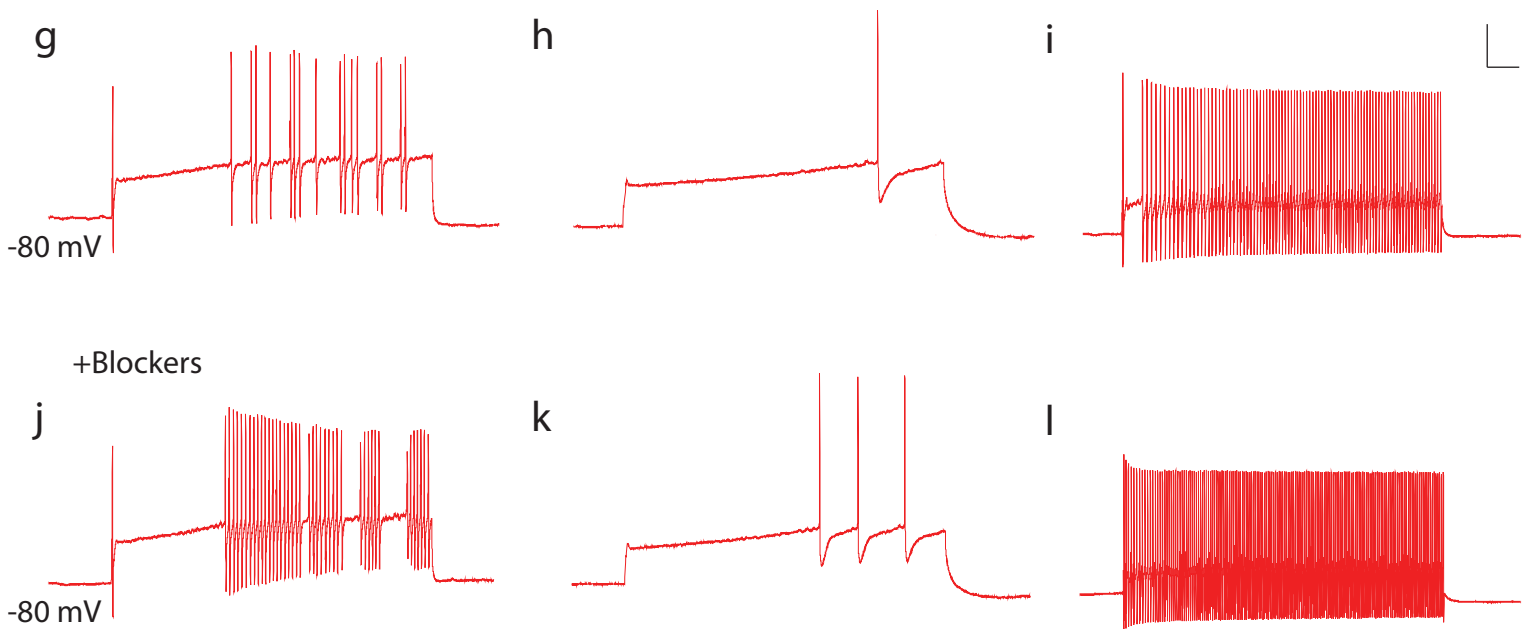

Supplement: Supplementary file 3 — Additional file 3: Figure S3. AP firing patterns of different DA neuronal subpopulations in the lateral and medial VTA without and with synaptic blockers. a-c) Examples of AP firing patterns of lateral VTA Type 1–3 neurons evoked by a 100 pA current step, without blockers. d-f) AP firing patterns of lateral VTA Type 1–3 neurons evoked by a 100 pA current step in the presence of synaptic blockers (Bicuculline 20 μM, D-AP5 50 μM, DNQX 10 μM, Sulpiride 500 μM, CGP55845 200 nM, Strychnine 1 μM). g-i) AP firing patterns of medial VTA Irregular, Delayed-Nonaccommodating, and High frequency DA neuron subpopulations at a 150 pA current step in the absence of synaptic blockers. j-l) AP firing patterns of medial VTA Irregular, Delayed-Nonaccommodating, and High frequency DA neuron subpopulations at 150 pA current step, with the above synaptic blockers. Scale bar, 20 mV, 100 ms. [file 13041_2019_511_MOESM3_ESM.pdf]

Lateral VTA

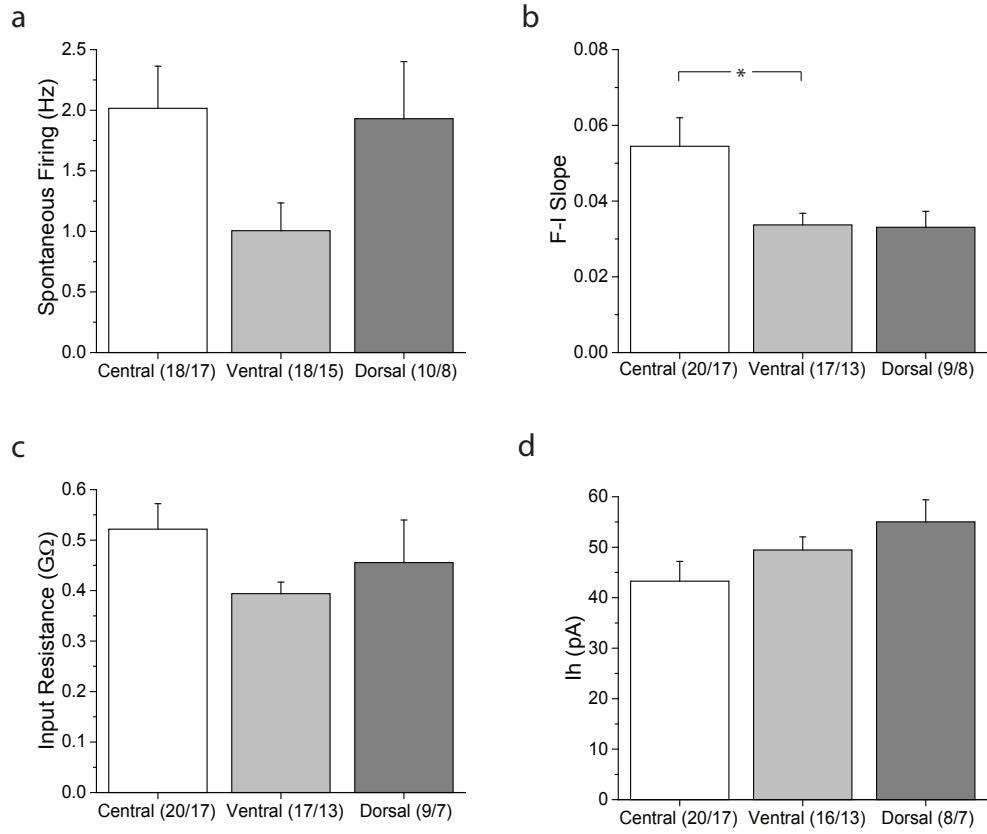

Medial VTA

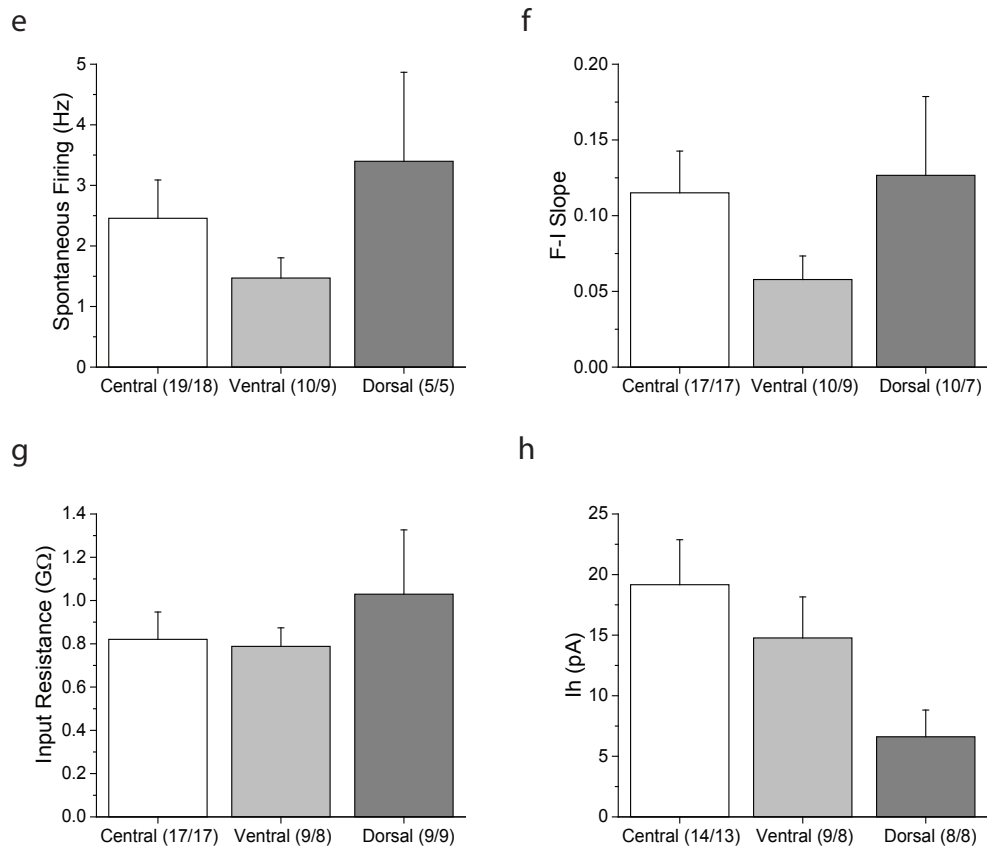

Supplement: Supplementary file 4 — Additional file 4: Figure S4. Electrophysiological properties of ventral-dorsal VTA DA neurons. a-d) Spontaneous firing frequency (a), F-I slope (b), input resistance (c), and Ih (d) in central, ventral, and dorsal subregions of the lateral VTA. e-h) Spontaneous firing frequency (e), F-I slope (f), input resistance (g), and Ih (h) in central, ventral, and dorsal subregions of the medial VTA. Numbers in parentheses reflect numbers of cells. DATA were collected from 33 SHAM and 25 SNI mice. [file 13041_2019_511_MOESM4_ESM.pdf]
